# Human immunodeficiency virus dynamics in secondary lymphoid tissues and the evolution of cytotoxic T lymphocyte escape mutants

**DOI:** 10.1093/ve/vead084

**Published:** 2024-01-11

**Authors:** Wen-Jian Chung, Elizabeth Connick, Dominik Wodarz

**Affiliations:** Department of Population Health and Disease Prevention, University of California, 856 Health Sciences Quad, Irvine, CA 92697, USA; Division of Infectious Diseases, Department of Medicine, University of Arizona, 1501 N. Campbell Ave, P.O. Box 245039, Tucson, AZ 85724, USA; Department of Ecology, Behavior, and Evolution, University of California San Diego, 9500 Gilman Drive, La Jolla, CA 92093, USA

**Keywords:** virus dynamics, mathematical model, compartmental model, virus evolution, follicular compartmen, extra-follicular compartment, CTL responses, CTL escape

## Abstract

In secondary lymphoid tissues, human immunodeficiency virus (HIV) can replicate in both the follicular and extrafollicular compartments. Yet, virus is concentrated in the follicular compartment in the absence of antiretroviral therapy, in part due to the lack of cytotoxic T lymphocyte (CTL)–mediated activity there. CTLs home to the extrafollicular compartment, where they can suppress virus load to relatively low levels. We use mathematical models to show that this compartmentalization can explain seemingly counter-intuitive observations. First, it can explain the observed constancy of the viral decline slope during antiviral therapy in the peripheral blood, irrespective of the presence of CTL in Simian Immunodeficiency Virus (SIV)-infected macaques, under the assumption that CTL-mediated lysis significantly contributes to virus suppression. Second, it can account for the relatively long times it takes for CTL escape mutants to emerge during chronic infection even if CTL-mediated lysis is responsible for virus suppression. The reason is the heterogeneity in CTL activity and the consequent heterogeneity in selection pressure between the follicular and extrafollicular compartments. Hence, to understand HIV dynamics more thoroughly, this analysis highlights the importance of measuring virus populations separately in the extrafollicular and follicular compartments rather than using virus load in peripheral blood as an observable; this hides the heterogeneity between compartments that might be responsible for the particular patterns seen in the dynamics and evolution of the HIV *in vivo*.

## 1. Introduction

The majority of HIV replication occurs in secondary lymphoid tissues, such as lymph nodes, spleen, or the gut-associated lymphoid tissue (Pantaleo et al. 1993). Within these tissues, virus replication is concentrated in the B cell follicles, with lower levels of replication observed in the extrafollicular (EF) compartments in the absence of antiretroviral therapy (Hufert et al. 1997; Tenner-Racz et al. 1998; Folkvord, Armon, and Connick 2005; Connick et al. 2007, 2014; Brenchley et al. 2012; Fukazawa et al. 2015; Ayala et al. 2017; Bronnimann, Skinner, and Connick 2018; Beck, Veenhuis, and Blankson 2019; Li et al. 2019; Xu, Ollerton, and Connick 2019). The follicular (F) compartments contain follicular CD4 T helper cells with higher permissiveness for viral replication than extrafollicular CD4 T cells and have a high concentration of extracellular virions on the surface of follicular dendritic cells. Both aspects promote viral spread. Another reason for the higher concentration of virus in the B cell follicles, however, is that these compartments are relatively immune privileged from CTL (Connick et al. 2007, 2014). Virus-specific CTLs are abundant in the extrafollicular compartment, to which CTL home and in which they are stimulated. Therefore, virus load is lower in the extrafollicular compartment, controlled by CTL, while virus load is significantly higher in the follicular compartments, where CTLs are substantially less abundant. This has been underlined with experiments in SIV-infected macaques, where the discrepancy in virus load between the two compartments is most pronounced in animals that show strong CTL-mediated control (Connick et al. 2007, 2014; Miles and Connick 2016) and where the distribution of virus across the two compartments is more equal in animals with weaker CTL responses or during the acute phase of infection, before CTL responses have fully matured (Connick et al. 2007, 2014; Miles and Connick 2016).

This unequal degree of CTL-mediated virus control in the follicular and extrafollicular compartments has implications for the evolutionary dynamics of CTL escape mutants. It is thought that CTL escape contributes to the loss of virus control over time as the disease progresses (Goulder and Watkins 2004). Point mutations occur in CTL epitopes, such that they are not recognized anymore by the responding CTL clone. A number of studies have pointed out that while CTL escape can occur rapidly during the initial stages of infection, the emergence of CTL escape occurs at a surprisingly slow rate in the longer term during chronic infection (Asquith et al. 2006; Davenport et al. 2008; Goonetilleke et al. 2009; Fryer et al. 2010; Roberts et al. 2015). Several reasons have been suggested to explain this phenomenon. In mathematical models, a broad CTL response directed against multiple epitopes of the virus, or a substantial fitness cost associated with escape, can render the evolution of escape more difficult to achieve, leading to a longer time until escape is observed (Althaus, De Boer, and Pascual 2008; Ganusov et al. 2011; van Deutekom, Wijnker, and de Boer 2013). Furthermore, mathematical models suggest that the latent viral reservoir can slow down evolutionary processes, and this might also contribute to a delayed emergence of CTL escape if the selective advantage of the escape mutant is not too high (Doekes, Fraser, and Lythgoe 2017). Another line of reasoning has been that lytic antiviral CTL responses in HIV infection are weaker than previously thought. While CTL responses have been shown to significantly suppress virus load, it is possible that these responses act predominantly in a non-lytic way, suppressing viral infection or replication rather than killing infected cells (Asquith et al. 2006; Seich Al Basatena et al. 2013). Mathematical analysis of escape evolution data showed that the relatively slow rate of escape emergence can be consistent with that notion (Asquith et al. 2006; Seich Al Basatena et al. 2013). This has been further underlined by studies showing that the rate of SIV decline during antiviral therapy was identical in in CTL-competent and CTL-depleted macaques (Klatt et al. 2010; Wong et al. 2010), arguing against the role of CTL in shortening the lifespan of infected cells. Similarly, the notion that CTLs act largely in a non-lytic rather than a lytic manner was also suggested as an explanation for the observed constancy of the estimated infected cell lifespan during antiviral therapy among people living with HIV, which was found to be around 1–2 days across different patients regardless of the disease stage/CD4 T cell count (Klenerman et al. 1996).

Here, we use mathematical models to show that the unequal distribution of CTL activity in the follicular and extrafollicular compartments of the secondary lymphoid tissues can significantly impact virus dynamics and the rate of CTL escape evolution in people living with HIV. First, compartmentalization can explain the observed constancy of the estimated infected cell lifespan during antiretroviral therapy, because estimates are based on peripheral blood measurements that are likely influenced most by virus in the follicular compartments, due to the high viral loads there. Second, compartmentalization can lead to significantly slower rates of CTL escape emergence compared to scenarios where overall virus load is the same, but cells and viruses mix extensively.

## 2. Basic virus dynamics in a compartmental model

We use a mathematical model that describes the dynamics between HIV and CTL responses in the follicular and extrafollicular compartments (Wodarz et al. 2018). The population of uninfected cells, infected cells, and CTL in the extrafollicular compartment are denoted by *X*_e_, *Y*_e_, and *Z*_e_, respectively. The corresponding populations in the follicular compartment are denoted by *X*_f_, *Y*_f_, and *Z*_f_, respectively. The time evolution of these populations is given by the following set of ordinary differential equations:


(1) $$\begin{aligned}&\frac{{{\mathrm{d}}{X_{\mathrm{e}}}}}{{{\mathrm{d}}t}} = {\lambda _{\mathrm{e}}} - \delta {X_{\mathrm{e}}} - {\beta _{\mathrm{e}}}{X_{\mathrm{e}}}{Y_{\mathrm{e}}},\nonumber\\ &\frac{{{\mathrm{d}}{Y_{\mathrm{e}}}}}{{{\mathrm{d}}t}} = {\beta _{\mathrm{e}}}{X_{\mathrm{e}}}{Y_{\mathrm{e}}} - a{Y_{\mathrm{e}}} - p{Y_{\mathrm{e}}}{Z_{\mathrm{e}}} - \eta {Y_{\mathrm{e}}} + \eta {Y_{\mathrm{f}}},\nonumber\\ &\frac{{{\mathrm{d}}{Z_{\mathrm{e}}}}}{{{\mathrm{d}}t}} = c{Y_{\mathrm{e}}} - b{Z_{\mathrm{e}}} - g{Z_{\mathrm{e}}} + h{Z_{\mathrm{f}}}{\mathrm{,\;}}\\ &\frac{{{\mathrm{d}}{X_{\mathrm{f}}}}}{{{\mathrm{d}}t}} = {\lambda _{\mathrm{f}}} - \delta {X_{\mathrm{f}}} - {\beta _{\mathrm{f}}}{X_{\mathrm{f}}}{Y_{\mathrm{f}}},\nonumber\\ &\frac{{{\mathrm{d}}{Y_{\mathrm{f}}}}}{{{\mathrm{d}}t}} = {\beta _{\mathrm{f}}}{X_{\mathrm{f}}}{Y_{\mathrm{f}}} - a{Y_{\mathrm{f}}} - p{Y_{\mathrm{f}}}{Z_{\mathrm{f}}} - \eta {Y_{\mathrm{f}}} + \eta {Y_{\mathrm{e}}},\nonumber\\ &\frac{{{\mathrm{d}}{Z_{\mathrm{f}}}}}{{{\mathrm{d}}t}} = g{Z_{\mathrm{e}}} - b{Z_{\mathrm{f}}} - h{Z_{\mathrm{f}}}.\nonumber\end{aligned}$$

In each compartment, uninfected cells are produced with a rate *λ*_e_ (*λ*_f_). These cells die naturally with a rate δ*X*_e_ (δ*X*_f_) and become infected with a rate *β*_e_*X*_e_*Y*_e_ (*β*_f_*X*_f_*Y*_f_). Infected cells die with a rate *aY*_e_ (*aY*_f_) and are killed by CTL with a rate *pY*_e_*Z*_e_ (*pY*_f_*Z*_f_). Infected cells migrate to the other compartment with a rate *ηY*_e_ (*ηY*_f_). CTLs become stimulated and expand in the extrafollicular compartment with a rate *cY*_e_. The rate of expansion is assumed to be not proportional to the number of CTL, since this has been shown to result in more stable and hence more realistic dynamics (Wodarz, Christensen, and Thomsen 2002). The CTLs migrate from the extrafollicular to the follicular compartment with a rate *gZ*_e_. In the follicular compartment, no CTL stimulation or expansion is assumed to occur, and CTLs migrate back into the extrafollicular compartment with a rate *hZ*_f_. In both compartments, CTLs are assumed to die with a rate *bZ*_e_ (*bZ*_f_).

### 2.1. Basic model properties

This model has been analyzed in some detail in Wodarz et al. (2018). For the limiting case *η* → 0, we can define the basic reproductive ratio of the virus in each compartment as *R*_0e_ = *λ*_e_*β*_e_/δ*a* and *R*_0f_ = *λ*_f_*β*_f_/δ*a*, respectively. If both are larger than one, the infection is established in both compartments. In the absence of immunity (*Z*_e_ = *Z*_f_ = 0), the following equilibrium is attained:


$$X_{\mathrm{e}}^* = \frac{a}{{{\beta _{\mathrm{e}}}}};{\mathrm{\;}}Y_{\mathrm{e}}^{\mathrm{*}} = \frac{{{\lambda _{\mathrm{e}}}}}{a} - \frac{\delta }{{{\beta _{\mathrm{e}}}}};{\mathrm{\;}}X_{\mathrm{f}}^* = \frac{a}{{{\beta _{\mathrm{f}}}}};{\mathrm{\;}}Y_{\mathrm{f}}^* = \frac{{{\lambda _{\mathrm{f}}}}}{a} - \frac{\delta }{f}.$$

For *η* > 0, an infection in both compartments is established if

$\delta \left( {a + \eta } \right)\left( {R_{0{\mathrm{f}}}^* + R_{0e}^* - {\mathrm{\;}}2} \right)\sqrt {{{\left( {{\beta _{\mathrm{f}}}{\lambda _{\mathrm{f}}} - {\mathrm{\;}}{\beta _{\mathrm{e}}}{\lambda _{\mathrm{e}}}} \right)}^2} + 4{\eta ^2}{\delta ^2}} > 0,$ where $R_{0{\mathrm{f}}}^* =$$\frac{{{\lambda _{\mathrm{f}}}{\beta _{\mathrm{f}}}}}{{\delta \left( {a + \eta } \right)}}$ and $R_{0{\mathrm{e}}}^* = \frac{{{\lambda _{\mathrm{e}}}{\beta _{\mathrm{e}}}}}{{\delta \left( {a + \eta } \right)}}$. Here, the expressions $R_{0{\mathrm{f}}}^*$ and $R_{0{\mathrm{e}}}^*$ are reproduction numbers for the two compartments that take into account virus spread within the compartment and loss of infected cells from death and emigration. The equilibrium expressions describing virus persistence in this case are too complicated to write down, but converge to the aforementioned expressions for low values of *η*, which is a biologically realistic assumption (see section 2.2). If the population of CTLs is added, they will expand as long as infected cells are present in the extrafollicular compartment, with the extent of the CTL expansion depending on the infected cell population size. The system then converges toward a stable equilibrium describing CTL-mediated virus control, which is again too complicated to write down. As with other CTL models (Nowak and Bangham 1996), equilibrium virus load is inversely proportional to the strength of the CTL response, which in this model is determined by the rate of CTL expansion or CTL responsiveness (parameter *c*) and the rate of CTL-mediated killing (parameter *p*).

### 2.2. Model parameters and data

Parameter values of the simulations are given in the figure legends. The lifespan of infected cells was set to be around 2 days (Perelson et al. 1996), and the remaining basic virus kinetic parameters were adjusted such that the basic reproductive ratio of the virus, *R*_0_, is around 8 (which corresponds to the estimated average value in people living with HIV (Ribeiro et al. 2010)) and such that populations persist robustly in stochastic Gillespie simulations explored in the section 3. After the initial model analysis, we will describe how model properties change if the assumed value of *R*_0_ is varied within clinically documented ranges. Beyond these values, model parameters are unknown. Therefore, we guide our parameter choices by experimental observations in SIV-infected macaques.

First, experimental data clearly show that CTLs are not present in significant numbers in the follicular compartment (Connick et al. 2007, 2014; Fukazawa et al. 2015; Li et al. 2017; Bronnimann, Skinner, and Connick 2018; Beck, Veenhuis, and Blankson 2019). CTLs home to the EF compartment, but the movement of CTL from the EF to the F compartment is minimal. Consequently, the follicular compartment is a relatively immune-privileged site in the context of CTL. Hence, we need to assume that the value of the parameter *g* (rate of CTL movement from EF to F) is relatively slow and that the parameter *h* (rate at which CTL home back to the EF from the F compartment) is relatively fast. It is further thought that the exchange of infected cells between the two compartments is limited, and therefore, we need to assume that the migration rate *η* is also relatively small. This arises from the observation that in chronic HIV/SIV infection, there is an unequal distribution of infected cells between the two compartments (Connick et al. 2007, 2014; Miles and Connick 2016), with the majority of infected cells being located in the follicular compartment. This is due to the limited CTL activity there, but even without CTL activity in the F compartment, this unequal distribution would not be observed if the migration rates of infected cells between the compartments were relatively large.

**Figure 1.**
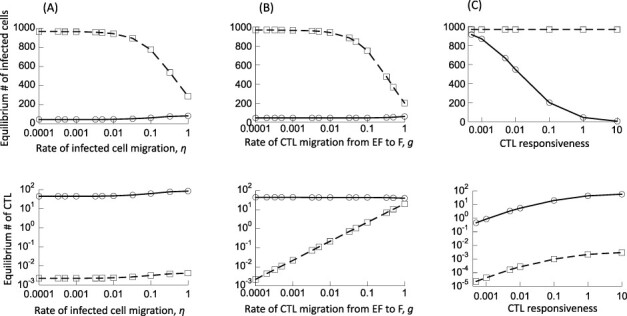
Basic properties of Model (1). The equilibrium number of infected cells (top row) and the equilibrium number of CTL (bottom row) are shown as a function of select model parameters. (A) Dependence on the migration rate of infected cells between compartments, *η*. (B) Dependence on the migration rate of CTL from the EF to the F compartment, *g*. (C) Dependence on the CTL responsiveness or rate of CTL activation, *c*. Dashed lines show the populations in the F compartment, and the solid line shows populations in the EF compartment. Baseline parameters are given as follows: *λ*_f_ = 500, *λ*_e_ = 500, *δ* = 0.01, *a* = 0.45, *β*_e_ = *β_f_* = 7 × 10^−5^, *p* = 0.05, *b* = 1, *c* = 1, *η* = 0.0001, *g* = 0.0001, and *h* = 1.

Figure 1A and B shows how the equilibrium number of infected cells in both compartments changes with an increase in the infected cell migration rate, *η* (Fig. 1A), and the rate of CTL movement from the EF to the F compartment, *g* (Fig. 1B), assuming a relatively strong CTL response. As these migration rates are increased from low to high, we see that the equilibrium number of infected cells in the F compartment declines continuously, first slowly, and then faster for larger migration rates. The more pronounced decrease in infected cells in the steady state in the F compartment for larger migration rates comes about because of a larger degree of mixing between the two compartments, which exposes an increasing number of infected cells from the follicular compartment to CTL-mediated activity. This is not observed in data from SIV-infected macaques, and therefore, these higher migration rates are not biologically relevant. A range of lower migration rates, however, is compatible with the lack of CTL-mediated control in the F compartment (Fig. 1A and B), because in this range, increasing the migration rate results in a minimal reduction in the equilibrium number of infected cells in the F compartment. As a baseline, we will assume that the migration rates are at the lower end, i.e. *η* = *g* = 10^−4^, and perform the initial analysis with these values. We subsequently investigate how variation in the parameters *η* and *g* (within ranges compatible with experimental data) influences the outcomes in the model. For *η* = *g* = 10^−4^, Fig. 1C shows that the model displays properties that are consistent with experimental observation in SIV-infected macaques (Connick et al. 2007, 2014; Miles and Connick 2016): as the strength of the CTL response (parameter *c*) is varied from low to high, we see a transition from a relatively even distribution of infected cells between the two compartments at equilibrium to a skewed distribution, in which steady state virus load in the EF compartment is low (controlled by CTL), while virus load in the F compartment is much higher, due to the lack of CTL-mediated activity there. This is consistent with our previous work (Wodarz et al. 2018).

### 2.3 Lytic CTL activity and the constancy of the infected cell lifespan during antiretroviral therapy (ART)

A key clinical/experimental observation in HIV and SIV dynamics has been the constancy of the virus decline rate in peripheral blood upon administration of antiviral therapy and hence the constancy of the estimated lifespan of infected cells (Ho et al. 1995; Wei et al. 1995; Klenerman et al. 1996; Perelson 2002). Regardless of whether the estimate is made early in HIV infection, when CTL responses are relatively strong or at more advanced stages of disease progression, and when CTL responses are weaker, the estimated infected cell lifespan has been found to be around 1–2 days (Klenerman et al. 1996). Even more striking is that CTL depletion in SIV-infected macaques have not resulted in a slower rate of virus decline in peripheral blood during antiviral therapy (Klatt et al. 2010; Wong et al. 2010). This has been difficult to interpret in the context of a lytic CTL response. It has been argued that CTLs act mostly in a non-lytic way by reducing viral spread through CTL-secreted signals (Asquith et al. 2006; Seich Al Basatena et al. 2013).

**Figure 2.**
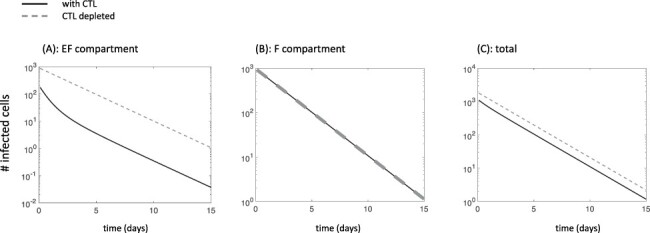
Simulation of ODE Model (1) during antiviral therapy (*β*_e_ = *β*_f_ = 0), in the presence (solid line) and the absence (dashed line) of CTL. (A) Dynamics in the extrafollicular compartment. (B) Dynamics in the follicular compartment. (C) Total dynamics, where populations are summed up over both compartments. Parameters were chosen as follows: *λ*_f_ = 500, *λ*_e_ = 500, *δ* = 0.01, *a* = 0.45, *β*_e_ = *β*_f_ = 7 × 10^−5^, *p* = 0.05, *b* = 1, *c* = 0.1, *η* = 0.0001, *g* = 0.0001, and *h* = 1. For CTL depletion, *c* = 0 and *p* = 0.

Our model only takes into account lytic CTL-mediated activity and ignores non-lytic suppression of virus replication by CTL. The reason is that we would like to investigate the dynamics under lytic activity, for which it has been more difficult to explain the evolutionary dynamics of the virus. Previous models of HIV dynamics with a lytic CTL response differentiated between two stages of infected cells, a cellular eclipse phase, and a virus production phase that is subject to CTL-mediated killing (van Deutekom, Wijnker, and de Boer 2013). This allowed the model to reproduce the observed behavior that the overall decline rate of infected cells during antiviral therapy remains relatively constant despite variations in the strength of CTL-mediated lysis. In our model, however, this distinction is not required to account for the constancy in the rate of infected cell decline observed in peripheral blood in the face of variations in the CTL response. It arises naturally from the uneven distribution of CTL in the extrafollicular and follicular compartments. Figure 2 uses our model to simulate the initial dynamics of infected cell decline during antiviral therapy (modeled by setting all infection rates to zero), brought about by the death of productively infected CD4 T cells. The infected cell decline is shown in both the presence and the absence of a CTL response. In the EF compartment, a clear difference is seen. The initial rate of infected cell decline is faster in the presence compared to the absence of CTL (Fig. 2A), due to the effect of CTL-mediated killing on the lifespan of infected cells. Over time, the rate of infected cell decline in the presence of CTL slows down, due to a reduction in the CTL population during treatment, when antigenic stimulation diminishes (not shown). In the F compartment, on the other hand, the rate of infected cell decline in the presence and absence of CTL is indistinguishable, due to the lack of significant CTL-mediated activity in this location (Fig. 2B). Because virus load is much higher in the F compared to the EF compartment (due to the lack of CTL activity), the rate at which the total number of infected cells (summed over both compartments) declines is dictated by the dynamics in F. In other words, the rate of decline of the overall number of infected cells is very similar in both the presence and the absence of the CTL response (Fig. 1C). Virus load measured in the peripheral blood likely reflects the sum of virus from the F and EF compartments. Hence, our model can account for the constancy of the virus decline slope in the peripheral blood irrespective of the strength of the CTL response, even though it assumes that CTL-mediated killing significantly suppresses the infected cell population (through action in the EF compartment). Note that we do not simulate the longer-term virus decline dynamics during antiviral therapy, since this would require a more complex model that tracks infected macrophages and latently infected cells (Perelson 2002); this is not within the current scope of the analysis.

In the Supplementary Materials, we show that the same dynamics occur if we chose higher migration rates *η* and *g*, as long as the difference in CTL-mediated activity and virus load between the two compartments is still substantial (see Fig. 1 for a discussion of this range). As can be seen in Supplementary Fig. S1, as the migration rates *η* and *g* are increased further (resulting in a lack of compartmentalization and more mixed populations), the overall rate of virus decline (summed over both compartments) becomes visibly dependent on the presence of the CTL response, which is at odds with experimental observations. Hence, a model with strong compartmentalization can better describe the experimentally observed constancy of the infected cell/virus decline rate during antiviral therapy in the context of efficient lytic CTL responses.

## 3. Evolutionary dynamics of CTL escape in a compartmental model

Here, we introduce a population of mutant virus, which is not recognized by CTL anymore. Again, this virus can replicate in both the extrafollicular and the follicular compartments, and the corresponding infected cells are denoted by *Y*_1e_ and *Y*_1f_, respectively. The escape variant is generated by mutation during wild-type virus replication with a rate *μ* and replicates itself with a rate *β*_1e_*X*_e_*Y*_1e_ (*β*_1f_*X*_f_*Y*_1f_). Note that the model does not include any backmutations. The resulting ordinary differential equations are given as follows.


(2) $$\begin{aligned}&\frac{{{\mathrm{d}}{X_{\mathrm{e}}}}}{{{\mathrm{d}}t}} = {\lambda _{\mathrm{e}}} - \delta {X_{\mathrm{e}}} - {X_{\mathrm{e}}}\left( {{\beta _{\mathrm{e}}}{Y_{\mathrm{e}}} + {\beta _{1{\mathrm{e}}}}{Y_{1{\mathrm{e}}}}} \right),\notag\\& \frac{{{\mathrm{d}}{Y_{\mathrm{e}}}}}{{{\mathrm{d}}t}} = {\beta _{\mathrm{e}}}{X_{\mathrm{e}}}{Y_{\mathrm{e}}}\left( {1 - \mu } \right) - a{Y_{\mathrm{e}}} - p{Y_{\mathrm{e}}}{Z_{\mathrm{e}}} - \eta {Y_{\mathrm{e}}} + \eta {Y_{\mathrm{f}}},\notag\\&\frac{{{\mathrm{d}}{Y_{1{\mathrm{e}}}}}}{{{\mathrm{d}}t}} = \mu {\beta _{\mathrm{e}}}{X_{\mathrm{e}}}{Y_{\mathrm{e}}} + {\beta _{1{\mathrm{e}}}}{X_{\mathrm{e}}}{Y_{{\mathrm{1e}}}} - a{Y_{1{\mathrm{e}}}} - \eta {Y_{{\mathrm{1e}}}} + \eta {Y_{1{\mathrm{f}}}},\notag\\&\frac{{{\mathrm{d}}{Z_{\mathrm{e}}}}}{{{\mathrm{d}}t}} = c{Y_{\mathrm{e}}} - b{Z_{\mathrm{e}}} - g{Z_{\mathrm{e}}} + h{Z_{\mathrm{f}}}\\&\frac{{{\mathrm{d}}{X_{\mathrm{f}}}}}{{{\mathrm{d}}t}} = {\lambda _{\mathrm{f}}} - \delta {X_{\mathrm{f}}} - {X_{\mathrm{f}}}\left( {{\beta _{\mathrm{f}}}{Y_{\mathrm{f}}} + {\beta _{1{\mathrm{f}}}}{Y_{1{\mathrm{f}}}}} \right),\notag\\&\frac{{{\mathrm{d}}{Y_{\mathrm{f}}}}}{{{\mathrm{d}}t}} = {\beta _{\mathrm{f}}}{X_{\mathrm{f}}}{Y_{\mathrm{f}}}\left( {1 - \mu } \right) - a{Y_{\mathrm{f}}} - p{Y_{\mathrm{f}}}{Z_{\mathrm{f}}} - \eta {Y_{\mathrm{f}}} + \eta {Y_{\mathrm{e}}},\notag\\&\frac{{{\mathrm{d}}{Y_{{\mathrm{1f}}}}}}{{{\mathrm{d}}t}} = \mu {\beta _{\mathrm{f}}}{X_{\mathrm{f}}}{Y_{\mathrm{f}}} + {\beta _{1{\mathrm{f}}}}{X_{\mathrm{f}}}{Y_{1{\mathrm{f}}}} - a{Y_{1{\mathrm{f}}}} - \eta {Y_{1{\mathrm{f}}}} + \eta {Y_{1{\mathrm{e}}}},\notag\\&\frac{{{\mathrm{d}}{Z_{\mathrm{f}}}}}{{{\mathrm{d}}t}} = g{Z_{\mathrm{e}}} - b{Z_{\mathrm{f}}} - h{Z_{\mathrm{f}}}.\notag\end{aligned}$$

CTL escape mutants can carry a fitness cost compared to the wild-type virus in the absence of CTL (Goulder and Watkins 2004), which is typically manifested in the parameter *β*. These mutants, however, tend to acquire compensatory mutations (Goulder and Watkins 2004) that greatly reduce this fitness cost. We do not model this process here, because it is not essential to the question under investigation and because this simplification limits the complexity of the model. We will hence assume a relatively small fitness cost of the escape mutant (1 per cent), expressed in a lower value of *β*_1e_ (*β*_1f_) compared to *β*_e_ (*β*_f_).

In this model, the CTL escape mutant and the wild-type virus compete. To better understand the competition outcome in this compartmental model, first consider the dynamics in the extrafollicular compartments only, ignoring the presence of the follicular compartments (this would correspond to *η* = 0 and *g* = 0). For the escape mutant to fixate and exclude the wild-type virus, the mutant has to essentially have an overall faster infection rate than the wild-type, which is given by the condition *β*_1e_ > *β*_e_(1 − *μ*). This is unlikely to hold because we assume that the existence of a 1 per cent fitness cost of the mutant and the mutation rate are relatively small (of the order of 10^–5^ per basepair per generation (Mansky and Temin 1995)). If this condition is not fulfilled, both the escape mutant and the wild-type virus will coexist at a level determined by the strength of the CTL response. The stronger the CTL response, the higher the abundance of the escape mutant relative to the wild-type virus. Although the escape mutants are advantageous in the presence of a sufficiently strong CTL response, exclusion of wild-type cannot occur if *β*_1e_ < *β*_e_(1 − *μ*). The reason is that the CTL response is not maintained in the absence of the wild-type virus, which allows the wild-type virus to invade the mutant population from low numbers due to its faster inherent replication rate without CTL.

**Figure 3.**
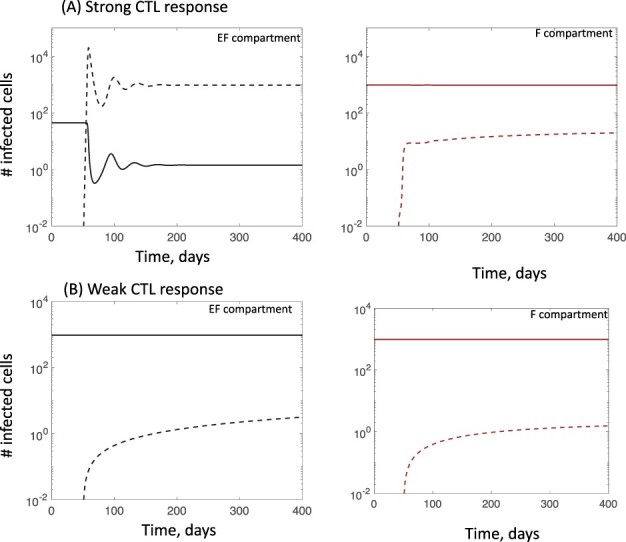
Outcomes in ODE Model (2), including a CTL escape mutant. These plots assume strong compartmentalization, i.e. infected cells move between compartments with a relatively slow rate, and CTLs enter the follicular compartment with a slow rate. Dynamics are shown for (A) a relatively strong CTL response and (B) a relatively weak CTL response, determined by the parameter *c*. Left and right graphs show dynamics in the extrafollicular and follicular compartments, respectively. Solid lines depict the dynamics of the wild-type virus, and dashed lines the dynamics of the CTL escape mutant. Parameters were chosen as follows: *λ*_f_ = 500, *λ*_e_ = 500, *δ* = 0.01, *a* = 0.45, *β*_e_ = β_f_ = 7 × 10^−5^, *β*_1e_ = *β*_1f_ = 0.99*β*_e_, *μ* = 2 × 10^−5^, *p* = 0.05, *b* = 1, *η* = 0.0001, *g* = 0.0001, and *h* = 1. For (A), *c* = 1. For (B), *c* = 10^−4^.

Next, consider the full two-compartment model under the same assumption about the infection rate of the CTL escape mutant relative to the wild type. Assuming low migration rates (*η* = *g* = 10^−4^) as stated in section 2.2, the relative abundance of the CTL escape mutant in the EF compartment again rises with an increasing strength of the CTL response (compare Fig. 3A and B). In contrast, in the F compartment, the escape mutant is maintained at a relatively low abundance, at a selection–mutation balance, regardless of the CTL strength (compare Fig. 3A and B). This is because the mutant is assumed to have an inherently lower replication rate and the CTL are assumed not to enter the F compartments at a significant rate (consistent with biological observations). While we assumed specific low values for the migration rates, the same results hold if the migration rates are somewhat higher, as long as CTL-mediated activity largely occurs in the EF compartment and the infected cells do not mix with a fast rate between compartments (Fig. 1). For a higher degree of mixing, the properties of the system converge to those of a single-compartment model.

### 3.1. Simulating the dynamics of CTL escape mutant invasion

We are interested in the time it takes for a CTL mutant to invade starting from a wild-type only population at equilibrium. Because mutant dynamics at low numbers are important in this respect, we perform stochastic Gillespie simulations (Gillespie 1976) of the ordinary differential equation ( ODE) model. Over time, the wild-type virus population generates mutants with a rate *μ*, and we determine the average time it takes for the mutant-infected cell population to reach 95 per cent of the total infected cell population in the extrafollicular compartment. In stochastic simulations with mutant generation, the mutant-infected cell population eventually reaches dominance in the EF compartments by selection or drift, depending on the strength of the CTL response relative to the assumed replicative fitness cost of the escape mutant. Due to the lack of any significant CTL-mediated selection pressure in the follicular compartment and the consequently larger infected cell population size, the CTL escape mutants practically never rise there in any realistic time frame, and the invasion of escape in the F compartment would not alter the dynamics in any meaningful way. Hence, we only consider escape mutant invasion in the EF compartment.

**Figure 4.**
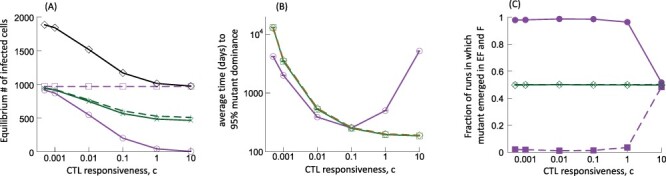
Stochastic evolutionary dynamics of CTL escape, as a function of the CTL responsiveness, *c*, according to Gillespie simulations of ODE Model (2) with relatively low migration rates *η* and *g*. Results are shown in purple lines across the different panels. These are compared to results from two control models, the mixed compartment control model (green lines) and the single-compartment control model (brown dashed line in panel B), as explained in the main text. (A) Equilibrium number of infected cells in the two compartments as a function of the CTL-responsiveness parameter *c*, as predicted by the ODEs. The number of cells in the EF and F compartments is shown in solid and dashed lines, respectively. This plot also shows in black the total equilibrium number of infected cells (summed over both compartments), which are identical to the number of infected cells in the single-compartment model (see the text for details). (B) The average time for the CTL escape mutant to reach 95 per cent of the infected cell population in the EF compartment. (C) Fraction of simulation runs in which the invading CTL escape mutant was generated in the F (dashed line) and the EF (solid line) compartment. Parameters were chosen as follows. For the compartmental model with low migration rates (purple lines): *λ*_f_ = 500, *λ*_e_ = 500, *δ* = 0.01, *a* = 0.45, *β*_e_ = *β*_f_ = 7 × 10^−5^, *β*_1e_ = *β*_1f_ = 0.99*β*_e_, *μ* = 2 × 10^−5^, *p* = 0.05, *b* = 1, *η* = 0.0001, *g* = 0.0001, and *h* = 1. For the mixed compartment control model (green lines), parameters were identical, but *η* = *g* = 1. For the single-compartment control model (brown dashed line): *λ* = 1000, *δ* = 0.01, *a* = 0.45, *β* = *7* × 10^−5^/2, *β*_1_ = 0.99*β*, *μ* = 2 × 10^−5^, *p* = 0.05, and *b* = 1. The value of *c* for both control models was adjusted such that total virus load was identical across all settings. The initial conditions for the Gillespie simulations were the equilibrium population sizes for the wild-type population, as predicted by the ODEs.

We start off by examining how the time to 95 per cent mutant invasion in the EF compartment depends on the strength of the CTL response, by varying the rate of CTL expansion, *c*. We use our baseline low migration rates (*η* = *g* = 10^−4^) for simulations and subsequently examine to what extent results change if the values of *η* and *g* are higher. As shown in Fig. 1C, at low values of *c*, the model shows relatively high equilibrium numbers of infected cells in both compartments, without a major difference between them. As the value of *c* is increased, the infected cell population in the steady state in the EF compartments is clearly reduced, while the number of infected cells in the follicular compartments remains high (due to the lack of significant CTL activity there). These trends are replotted in Fig. 4A (purple line) for reference. Figure 4B (purple line) shows that the average time to mutant invasion in the EF compartment has a minimum for an intermediate strength of the CTL response, *c*. This is intuitive and expected. For weak CTL responses, virus load is high in both compartments and the selection pressure is low. While mutants are generated with a relatively fast rate, their ability to rise at the expense of the wild-type virus is limited. For strong CTL responses, there is a high selection pressure in the EF compartment, which facilitates escape mutant invasion. Due to the low virus loads in the EF compartment, however, the probability to generate a mutant is low, which contributes to the increased invasion times. Mutants can be more readily produced in the F compartment, where virus load is higher. However, due to the limited CTL activity in the F compartment, the generated escape mutants are unlikely to rise to significant levels. They are more likely to go extinct instead, especially because they are assumed to carry an intrinsic fitness cost. For intermediate rates of CTL proliferation, virus load in the EF compartment is intermediate, providing a higher chance to generate the CTL escape mutant, while selection still favors the escape mutant to a sufficient extent. This results in the shortest mutant invasion times.

We determined the contribution of the EF and F compartments to mutant evolution, by tracking the origin of the escape mutants in the computer simulations. When the total mutant-infected cell fraction in the EF compartment reached 95 per cent, we determined in which compartment the largest escape clone originated and thus recorded the fraction of simulation runs in which the largest escape clone originated in the EF and F compartments, respectively. The mutants are mostly generated in the EF compartment over a wide range of CTL expansion rates (Fig. 4C, purple lines). The reason is that in the F compartment, very few CTLs are present and the escape mutant is assumed to carry an intrinsic fitness cost. Hence, the mutant is not likely to sufficiently rise in the follicles and move to the EF compartment. For very strong CTL responses, however, escape mutants originate more frequently in the F compartment. In this parameter regime, the CTL response essentially eliminates productive virus from the EF compartment, which is only maintained there by influx from the follicles. In this regime, there is thus an increased chance that escape mutants are produced in the follicle and subsequently move to the extrafollicular compartment rather than originating in the EF compartment where virus replication is very limited. Consequently, mutant invasion times are relatively long (Fig. 4B).

### 3.2. Effect of the compartmental structure

To determine how the compartmental structure (with low migration rates, *η* = *g* = 10^−4^) determines the CTL escape mutant invasion times in our model, we have to compare the above-described results to control scenarios. The first control scenario assumes strong mixing of populations between the two compartments such that the infected cell population is close to being distributed equally among the F and EF compartments (Fig. 4A, green lines). We refer to this as the ‘mixed compartment control model’. The second control scenario is a ‘single-compartment control model’, which lacks compartmental structure altogether. A complication is that changing the migration rates of cells or eliminating the compartmental structure leads to different equilibrium infected cell population sizes in the models. At the same time, it is known that population size is a major determinant of mutant invasion times in evolutionary models (Kimura and Ohta 1969). To determine the effect of the compartmental structure (rather than differences in the infected cell population size) on escape mutant invasion times, we adjusted the rate of CTL expansion in both the mixed compartment control model and in the single-compartment control model, such that the equilibrium number of infected cells is identical compared to the original model, which assumed low migration rates (*η* = *g* = 10^−4^). Thus, for each value of *c* in the low migration compartmental model (Fig. 4, purple line), we adjusted the value of *c* in both control scenarios such that the equilibrium number of infected cells is identical across all scenarios considered. We note that the escape mutant invasion times in both control scenarios are identical (Fig. 4B green and brown dashed lines), which makes intuitive sense because a high degree of mixing between compartments results in dynamics that converge to those observed for a single-compartment model.

For more efficient CTL responses (higher value of *c*), Fig. 4B shows that the compartmental structure strongly delays the time of mutant invasion compared to the single-compartment control model (or the mixed compartment control model). The reason is that with strong compartmentalization, the low selection pressure in the F compartment makes it unlikely for mutants to rise there and take over the EF compartment; at the same time, the strong CTL-mediated virus control in the EF compartment results in only infrequent mutant production in that location. In contrast, without compartmentalization, the CTL can attack all the infected cell population and total virus load is intermediate (Fig. 4A). This leads to overall faster mutant production, coupled by relatively strong selection.

For weaker CTL responses, the delay in mutant invasion brought about by compartmentalization is not observed anymore. In fact, the mutant invasion times are slightly shorter in simulations with low migration rates and strong compartmentalization compared to the control scenarios without compartmentalization (Fig. 4B). The reason is that for weak CTL responses, there is little CTL-mediated virus control in the EF compartment, and hence, the distribution of virus across the two compartments is more equal despite the low migration rates (which is consistent with experimental data). In this case, we are comparing the invasion of mutants in the EF compartment (with only weak CTL activity) to the invasion of the mutant in a mixed system, which essentially includes all infected cells, not just the ones in the EF compartment. Hence, the total population size relevant for mutant invasion is smaller for the compartmental model with low migration rates (because the F compartment is essentially separated from the EF compartment), which accounts for the comparatively shorter invasion times of the low migration compartmental model if CTL responses are weak.

### 3.3. Further parameter dependencies

So far, we have assumed particular parameter values that are consistent with experimental measurements or that give rise to model properties that are consistent with experimental observations. In this section, we re-examine the simulations if these parameter values are varied within biologically reasonable constraints.

**Figure 5.**
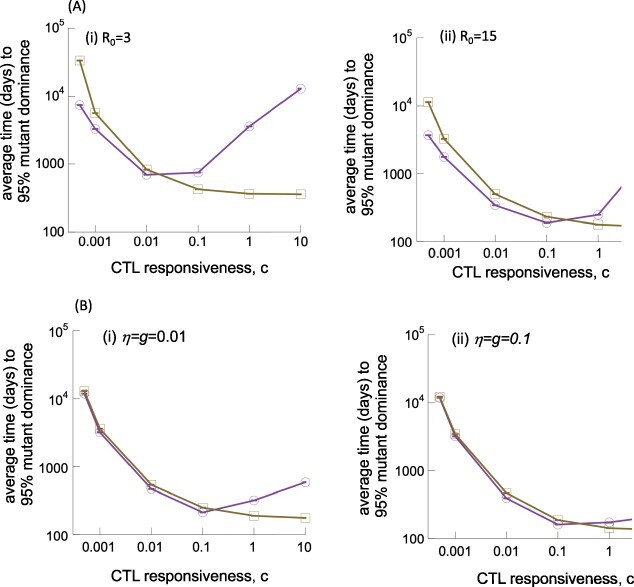
Effect of parameter variations on the time to 95 per cent mutant dominance in the EF compartment, according to Gillespie simulations of Model (2). Results from the two-compartment model with low migration rates (purple line) are compared to those from the single-compartment control model (brown line). These are the same kinds of simulations as presented in Fig. 4B. (A) Results for (i) a lower and (ii) a higher value of the basic reproductive ratio of the virus, *R*_0_. (B) Results for higher migration rates *η* and *g* that are still compatible with the experimentally observed compartmentalization of virus load (see the text for details). Parameters were chosen as follows: for (A), *λ*_f_ = 500, *λ*_e_ = 500, *δ* = 0.01, *a* = 0.45, *μ* = 2 × 10^−5^, *p* = 0.05, *b* = 1, *η* = 0.0001, *g* = 0.0001, and *h* = 1. (i) *β*_e_ = *β*_f_ = 2.7 × 10^−5^ and *β*_1e_ = *β*_1f_ = 0.99*β*_e_ and (ii) *β*_e_ = *β*_f_ = 1.35 × 10^−4^ and *β_1_*_e_ = *β*_1f_ = 0.99*β*_e_. For (B), *λ*_f_ = 500, *λ*_e_ = 500, *δ* = 0.01, *a* = 0.45, *β*_e_ = *β*_f_ = 7 × 10^−5^, *β*_1e_ = *β*_1f_ = 0.99*β*_e_, *μ* = 2 × 10^−5^, *p* = 0.05, *b* = 1, and *h* = 1. Values of *η* and *g* as indicated in plots.

First, consider the basic reproductive ratio of the virus, *R*_0_, which was found on average to be around eight for people living with HIV (Little et al. 1999; Ribeiro et al. 2010) as well as for SIV-infected macaques (Nowak et al. 1997), using basic virus dynamics models for parameter estimation. However, clinical data indicate a significant spread of *R*_0_ values among different people living with HIV (Ribeiro et al. 2010), and models including time delays between infection and virus production can lead to increased *R*_0_ estimates (Nowak et al. 1997; Little et al. 1999). Hence, we repeated our analysis assuming *R*_0_ = 3 on the lower side and *R*_0_ = 15 on the higher side (both within the observed ranges), by varying the virus infection rate of cells (Fig. 5). Here, we only compare the low migration compartmental model (*η* = *g* = 10^−4^) to the single-compartment control model (and omit the mixed compartmental control model because its properties converge to those of the single-compartment model). As seen in Fig. 5A, the trends remain robust in the context of different *R*_0_ values of the virus. The delay in escape mutant dominance for stronger CTL responses is more pronounced for the lower value of *R*_0_ and less pronounced for higher values of *R*_0_.

Next, we consider the migration rate of infected cells (*η*) and the rate of CTL movement from EF to F (*g*). We used a relatively low but arbitrary migration rate in simulations so far, because this parameter choice recapitulates the experimental finding that virus load is relatively evenly distributed across the compartments for weak CTL responses, but skewed toward the F compartment for stronger CTL responses (Fig. 1). This property, however, is also observed if somewhat higher migration rates *η* and *g* are assumed (Fig. 1A, B), so we need to examine how higher migration rates that are still qualitatively consistent with experimental observations affect our results. Thus, we repeated the simulations with *η* = *g* = 0.01 and *η* = *g* = 0.1 (Fig. 5B). As seen in Fig. 1B and C, while the equilibrium number of infected cells declines with the migration rates *η* and *g*, the decline only becomes more pronounced around *η* ≥ 0.1 and *g* ≥ 0.1. This means that CTL-mediated reduction of virus load in the F compartment occurs for these larger migration rates, which is not consistent with experimental observations. This is the rational for not running the simulation beyond *η* = 0.1 and *g* = 0.1. Figure 5B shows that our previously described trend for the average invasion time of CTL escape mutants holds true for the larger migration rates used here (and still consistent with experimental observation), although the delayed invasion time as a result of compartmental structure becomes less pronounced for larger migration rates.

Throughout our analysis, we assumed that the migration of infected cells between the two compartments is symmetrical, i.e. infected cells move from the EF to the F compartment and from the F to the EF compartment with a rate *η*. If the infected cell migration rates between the two compartments are asymmetric, it is likely that the migration rate from the F to the EF compartment is dominant. Imaging has shown that infected cells in the EF compartment of intestinal tissues are often located close to the B cell follicle (Connick et al. 2014), indicating recent immigration of the infected cell from the F compartment before being removed by CTL. We have explored computer simulations assuming that infected cells only migrate from the F to the EF compartment and that there is zero migration in the opposite direction (Supplementary Fig. S2). Results are almost unchanged compared to the simulation assuming symmetric migration. The reason is that the migration of infected cells from the F to the EF compartment is the crucial component in these dynamics. First, it can ensure that productive infection in the EF compartment is maintained in the face of strong CTL responses. Second, mutant fixation in the EF compartment is influenced only by migration if mutant cells that have been generated in the follicles migrate to the EF compartments and thus contribute to mutant invasion there.

## 4. Discussion and conclusion

Mathematical models have made major contributions to our understanding of HIV dynamics (Perelson 2002). Most of these were possible with simplified models that treated the HIV population within an individual as a single, well-mixed system. In some studies, spatial models were used to explore virus spread under more complex assumptions (Frost et al. 2001; Seich Al Basatena et al. 2013). The explicit consideration of virus replication in extrafollicular and follicular compartments, however, has been largely missing. At the same time, recent data indicate that this compartmentalization might be an important factor for determining the extent to which CTL can control the infection. The follicular compartment represents a relatively immune-privileged site in the context of CTL, where the infected cells are shielded from CTL-mediated activity. Failure of CTL to accumulate in the follicles has been observed in people living with HIV (Connick et al. 2007). In SIV-infected macaques, the presence of strong CTL responses has been shown to result in an unequal distribution of the virus across the two compartment types, with high virus loads in the follicles and low virus loads in the extrafollicular compartments, controlled by CTL (Connick et al. 2014). In contrast, the virus population is distributed more equally across compartments in animals with weak CTL responses or in the acute phase of the infection before CTL responses have emerged (Connick et al. 2014). These observations have been replicated in a mathematical modeling study, which investigated compartmental dynamics under different assumptions (Wodarz et al. 2018).

Here, we have built on this modeling work and have shown that the compartmental structure of the secondary lymphoid tissue has a significant impact on the *in vivo* dynamics of HIV that can explain seemingly counter-intuitive results. First, the model can account for conflicting observations that demonstrate the importance of CTL-mediated lysis for limiting virus load and at the same time show a lack of correlation between the strength of the CTL response and the rate of virus decline during antiviral therapy in people living with HIV (Klenerman et al. 1996) or in SIV-infected macaques (Wodarz et al. 2018). According to the model, the virus decline observed in the blood represents mostly the decline of infected cells in the follicular compartments, because this population is not significantly suppressed by CTL and is therefore dominant, even though CTL-mediated lysis is assumed to strongly suppress virus load in the extrafollicular compartment, limiting the total amount of virus in the host. It would be instructive to measure the rate of infected cell decline during antiviral therapy in the presence and absence of CTL separately for the extrafollicular and the follicular compartments. According to our theory, CTL depletion should significantly affect the rate of infected cell decline in the extrafollicular, but not in the follicular compartments.

Second, in the presence of relatively strong CTL responses, the compartmental model predicts significantly longer times for the emergence of CTL escape mutants compared to control models in which cells mix more readily between the compartments or in which the compartmental structure is lacking. The reason is that in the extrafollicular compartments, where CTL-mediated selection pressure is strong, the chances to generate an escape mutant are small due to low virus load. In the follicular compartment, where virus load is relatively high, escape mutants can be readily generated, but they are unlikely to invade given the lack of a selective advantage in this location. Hence, this results in longer emergence times compared to a model without the compartmental structure but the same total virus load. This adds to the explanations for the observed slow emergence of CTL escape in untreated people living with chronic HIV, reviewed in the Introduction section. It is interesting to note that the compartmental structure leads to the longest delay in CTL escape mutant emergence compared to mixed control models for relatively strong lytic CTL responses. This is in contrast to the notion that unexpectedly long times until escape mutant emergence are indicative of a relatively weak CTL response or predominantly non-lytic CTL activity (Asquith et al. 2006; Seich Al Basatena et al. 2013).

An important extension of the mathematical work reported here is the broader analysis of viral evolutionary dynamics in the presence of compartmentalization. While there is a clear difference in viral fitness between the two compartment types for CTL escape mutants, the relative fitness of mutants in the follicular and extrafollicular compartments is likely the same for other viral variants that do not contribute to CTL escape. Previous evolutionary work, however, has shown that variation in population sizes across demes can itself change mutant invasion dynamics (Yagoobi and Traulsen 2021). Moreover, differences in the stochastic fluctuations in population sizes between the follicular and extrafollicular compartments, which can be brought about by the variation in CTL-mediated activity, can further influence the evolutionary dynamics of the virus (Parsons and Quince 2007). These effects can be quantified by an extension of the mathematical framework presented here.

The formulation of the CTL dynamics in our model is characterized by some uncertainty. The CTL expansion term is phenomenological, simply assuming that the virus-specific CTL population expands at a rate proportional to the amount of their antigen present, *cY*. Other models have assumed true proliferation of the CTL population in response to antigen, where the expansion term is given by *cYZ* (Nowak and Bangham 1996). This, however, leads to less stable dynamics with strong oscillations that are not seen in CTL responses *in vivo*. Other models included a certain number of pre-programmed CTL divisions following antigenic stimulation (Antia, Ganusov, and Ahmed 2005; Wodarz and Thomsen 2005). Based on the comparison of the properties of these models, we do not expect that such differences would lead to qualitatively different results in the context of our study. Another assumption we made in our model was that CTL can only become stimulated by antigen in the EF compartments and not in the F compartments. This is based on the notion that CTLs home to the EF compartments and limit their presence in the F compartments (Connick et al. 2007). In the context of engineered CTL that can home to the F compartment (Pampusch et al. 2022), some degree of antigenic stimulation was observed in the follicles. We do not expect the results to change if the rate of CTL stimulation in the F compartment is relatively low and the migration of CTL from the F to the EF compartment is relatively large. If, however, significant CTL expansion and activity were assumed to occur in the F compartments, then the model properties would likely become different because this would alter and reduce the differences in virus load across the two compartments.

In conclusion, our models suggest that the compartmentalization of virus replication in the secondary lymphoid tissues, with relatively strong CTL-mediated virus control in the extrafollicular compartment, coupled with high virus loads and limited CTL activity in the follicles, can fundamentally influence the dynamics of HIV infection. It can explain the lack of variation in the overall rate of virus decline during antiviral therapy in the peripheral blood, even if CTL-mediated lysis contributes significantly to virus control and if patients differ in the degree of CTL-mediated lytic activity (Pampusch et al. 2022). It can further provide an explanation for the unexpectedly long CTL escape emergence times that have been observed in chronic HIV infection (Asquith et al. 2006; Davenport et al. 2008; Goonetilleke et al. 2009; Fryer et al. 2010; Roberts et al. 2015), even under the assumption that CTL-mediated lysis is a significant contributor to virus control. Therefore, it might be important to investigate more closely the heterogeneity in virus dynamics between the follicular and extrafollicular compartments when studying the dynamics and evolution of HIV *in vivo*, rather than solely concentrating on plasma virus loads measured in patients.

## Supplementary Material

vead084_Supp

## Data Availability

This paper is based on mathematical models and does not contain novel data.
