# Correction to: Human immunodeficiency virus dynamics in secondary lymphoid tissues and the evolution of cytotoxic T lymphocyte escape mutants

**DOI:** 10.1093/ve/veae052

**Published:** 2024-07-18

This is a correction to: Wen-Jian Chung, Elizabeth Connick, Dominik Wodarz, Human immunodeficiency virus dynamics in secondary lymphoid tissues and the evolution of cytotoxic T lymphocyte escape mutants, *Virus Evolution*, Volume 10, Issue 1, 2024, vead084, https://doi.org/10.1093/ve/vead084.

The grant number included in the Funding section has been corrected.

