# Supplementary material for: Human immunodeficiency virus dynamics in secondary lymphoid tissues and the evolution of cytotoxic T lymphocyte escape mutants
Source: Virus Evol. 2024 Jan 11;10(1):vead084. doi: 10.1093/ve/vead084 (PMC10956502; doi:10.1093/ve/vead084)
Supplement: vead084_Supp [file vead084_supp.zip › suppl_data/Supplementary Materials-clean.pdf]

# Supplementary Materials:
# further details about the mathematical models

## 1. Single-Compartment model of virus dynamics and CTL escape

The main text defines compartmental models of virus dynamics to study the evolution of cytotoxic T lymphocyte (CTL) escape mutants by human immunodeficiency virus (HIV). In particular, we distinguish between the follicular and extrafollicular compartments in the secondary lymphoid tissues. The results obtained for these models are compared to those seen in a corresponding single-compartment control model, which is outlined as follows.

We denote the populations of uninfected cells, infected cells, and CTL by *x*, *y*, and *z*, respectively. The time evolution of these populations is given by the following set of ordinary differential equations:

$$\frac{dX}{dt} = \lambda - dX - \beta XY,$$

$$\frac{dY}{dt} = \beta XY - aY - pYZ,$$

$$\frac{dZ}{dt} = cY - bZ.$$

Similar models have been widely used in the literature, e.g. [1-4]. Target cells are produced with a rate $\lambda$, die with a rate $dX$, and become infected by virus with a rate $\beta XY$ (assuming that virus is in a quasi-steady state). Infected cells are characterized by a basic death rate $aY$, and are killed further by CTL with a rate $pYZ$. The CTL population expands with a rate $cY$ and dies with a rate $bZ$.

In the absence of the CTL, the virus population establishes a persistent infection if its basic reproductive ratio $R_0=\beta\lambda/da>1$. The system then converges to a stable equilibrium, given by $X^{(1)}=a/\beta, Y^{(1)}=\lambda/a - d/\beta$, $Z^{(1)}=0$. If $c>0$, the CTL response expands

in the presence of the infection. In this case, the populations converge towards the following stable equilibrium.

$X^{(2)} = \frac{a\,b\,\beta - c\,d\,p + \sqrt{(a\,b\,\beta - c\,d\,p\,)^2 + 4b\beta^2\lambda cp}}{2b\beta^2}$,

$Y^{(2)} = \frac{b(\beta X^{(2)} - a)}{cp}$,

$Z^{(2)} = \frac{\beta X^{(2)} - a}{p}$.

Next, we incorporate a CTL escape virus strain into the model, described by the subscript "1"; the infected cells are hence denoted by $Y_1$. The modified ODEs are given as follows:

$\frac{dX}{dt} = \lambda - dX - \beta XY - \beta_1 XY_1$,

$\frac{dY}{dt} = \beta XY(1-\mu) - aY - pYZ$,

$\frac{dY_1}{dt} = \mu\beta XY + \beta_1 XY_1 - aY_1$,

$\frac{dZ}{dt} = cY - bZ$.

The CTL escape virus strain is characterized by its specific rate of infection, $\beta_1$, and is not killed by the CTL population. It is produced during infection events with a probability $\mu$. We assume that the $R_0$ of both virus strains is greater than unity, and that the CTL population expands. In this case, two outcomes are possible. If $\beta 1 > \beta(1-\mu)$, the escape mutant will fixate (with the wild-type virus going extinct). Because the wild-type virus goes extinct and the escape mutant does not stimulate the CTL response, the CTL population also goes extinct in this model. The equilibrium is hence given by

$X^{(3)}=a/\beta_1$, $Y^{(3)}=0$, $y1^{(3)}=\lambda/a - d/\beta_1$, $Z^{(3)}=0$. Otherwise, the escape mutant coexists with the wild-type virus and the CTL response against the wild-type virus also persists, i.e. all population sizes are greater than zero. The equilibrium expressions for this outcome are given by the solution of a third degree polynomial and are thus not specified here.

## 2. Effect of migration rates on the rate of infected cell decline during anti-viral therapy in the compartmental model

In the main text, the compartmental model was used to study the rate of infected cell decline in the EF and F compartments, as well as when virus load from both compartments are combined (which would correspond to measurements in peripheral blood). This was done by assuming an arbitrary low migration rate that is consistent with experimental observation ($\eta$ =$g$=$10^{-4}$, see section “Model parameters and data” in main text). Here we explore how these dynamics change if the migration rates are increased up to values for which the system can be considered

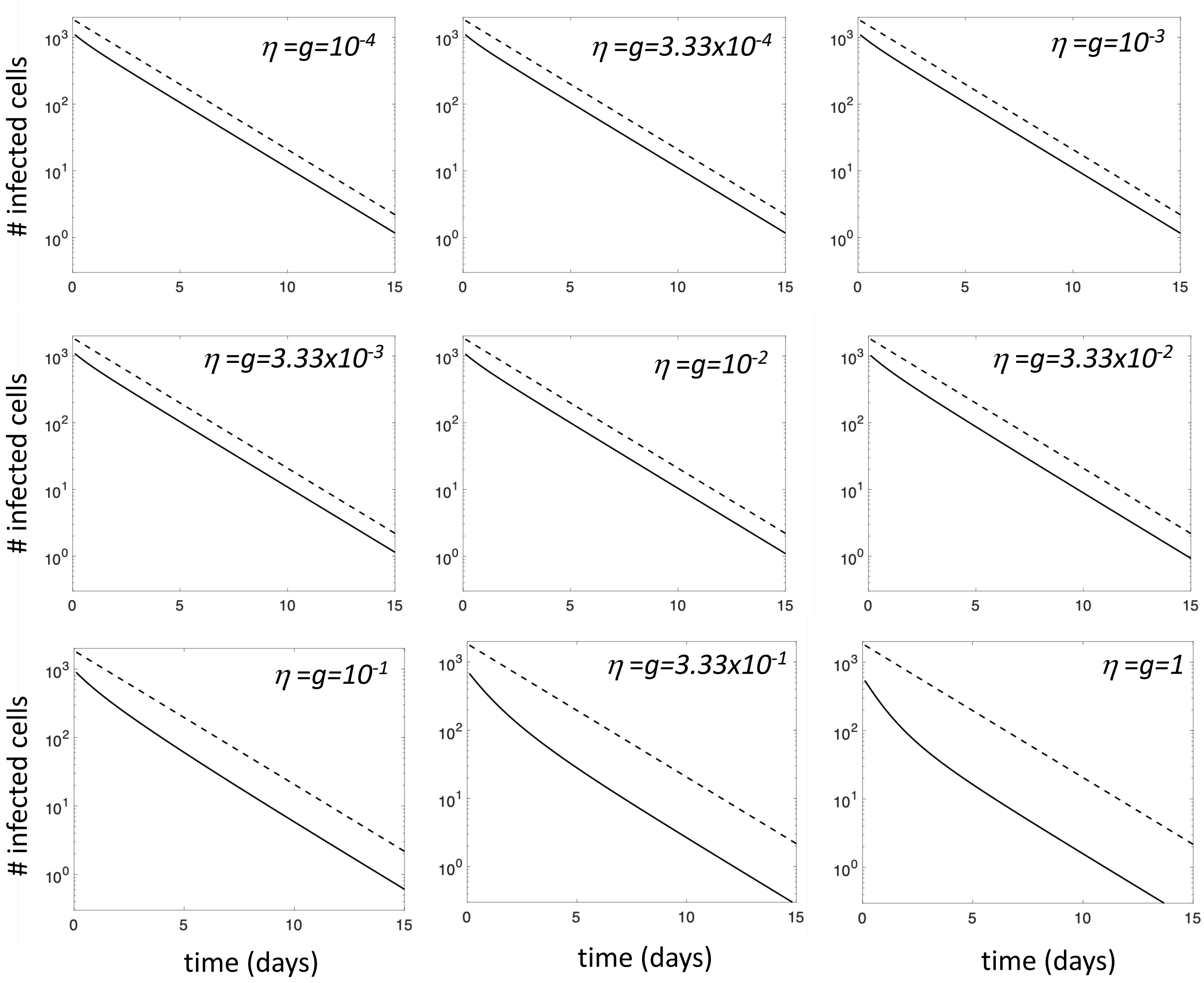


**Figure S1.** Simulated rate of total infected cell decline during anti-retroviral therapy in the presence (solid line) and absence (dashed line) of CTL, according to model (1). The sum of infected cells across the EF and F compartment is plotted. This is the same as Figure 2C in main text, but we vary the values of the migration rates $\eta$ and $g$, as indicated in the plots.

well-mixed. Results are shown in Figure S1. As can be seen in these plots, larger migration rates show a progressively more pronounced effect of CTL on the initial decline rate of the total virus population summed over both compartments.

## 3. Effect of unequal infected cell migration rates in the model

In the main analysis, we assumed that the migration rate of infected cells between the two compartments was identical in both directions. It might be more realistic to assume that infected cells move predominantly from the F to the EF compartment, thus contributing to the persistent productive virus replication in the face of strong CTL responses in the extrafollicular compartment. In Figure S2, we re-ran the simulations from Figure 4B in the main text under the assumption that infected cells only migrate from the F to the EF compartment (and not in the opposite direction). As can be seen in this plot, this change does not change our results or conclusions. Reasons are discussed in the main text.

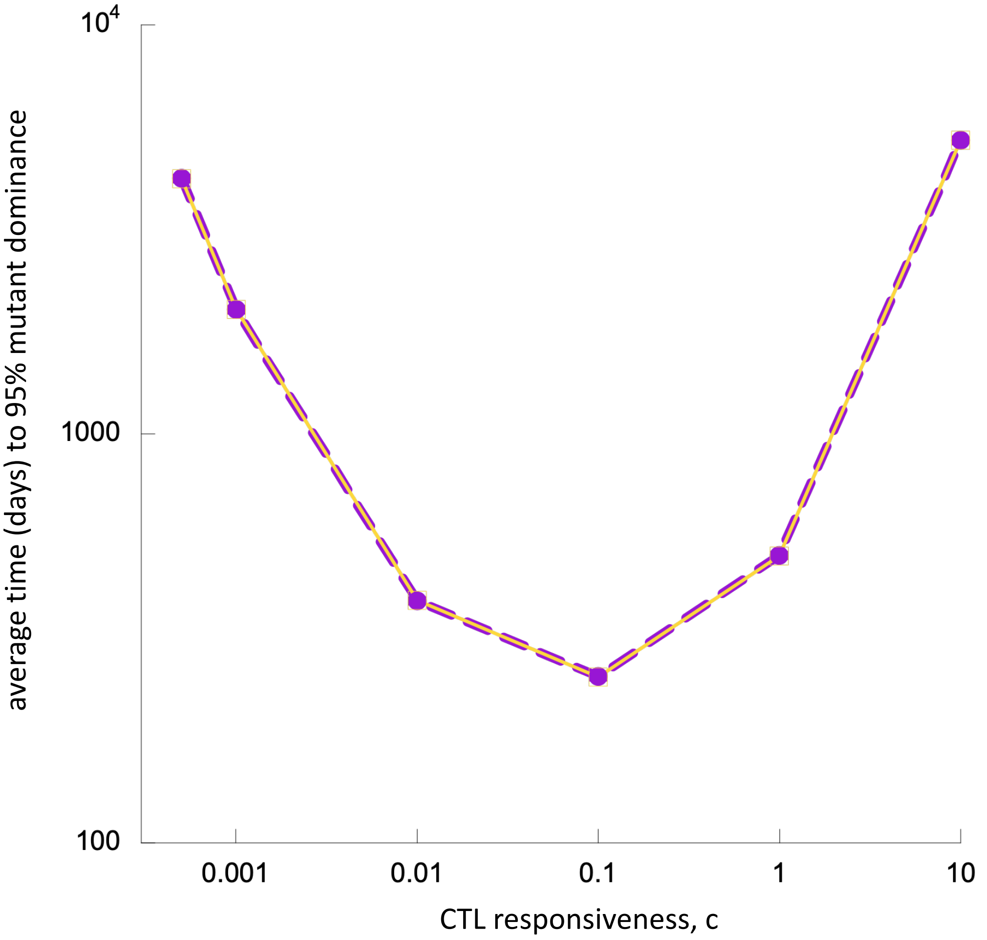


**Figure S2.** Effect of assuming uni-directional migration of infected cells from the F to the EF compartment on the stochastic behavior of model (2). Plotted is the average time for the CTL escape mutant to reach 95% of the infected cell population in the EF compartment, using the compartmental model with low migration rates. The purple (dashed) line is identical to the purple line in Figure 4B (identical parameters). The yellow (solid) line is a version of the simulation in which infected cell migration only occurs from the EF to the F compartment (with rate $\eta = 10^{-4}$). All other parameters are the same as for the purple line in Figure 4B, main text. Error bars (standard errors) are plotted, but are too small to see due to the large number of simulation repeats.

## References

[1] Nowak, M.A. & May, R.M. 2000 *Virus dynamics. Mathematical principles of immunology and virology.*, Oxford University Press.
[2] Perelson, A.S. 2002 Modelling viral and immune system dynamics. *Nature Rev Immunol* **2**, 28-36.
[3] Perelson, A.S. & Ribeiro, R.M. 2013 Modeling the within-host dynamics of HIV infection. *BMC Biol* **11**, 96. (doi:1741-7007-11-96 [pii]
10.1186/1741-7007-11-96).
[4] Wodarz, D., Christensen, J.P. & Thomsen, A.R. 2002 The importance of lytic and nonlytic immune responses in viral infections. *Trends Immunol* **23**, 194-200.
